# *Argas persicus* and *Carios vespertilionis* Ticks Infesting Ducks, Domestic Fowls and Bats in Pakistan: First Report on Molecular Survey and Phylogenetic Position of *Borrelia anserina*

**DOI:** 10.3390/vetsci10100628

**Published:** 2023-10-20

**Authors:** Hafsa Zahid, Abdulaziz Alouffi, Mashal M. Almutairi, Muhammad Ateeq, Tetsuya Tanaka, Shun-Chung Chang, Chien-Chin Chen, Abid Ali

**Affiliations:** 1 Department of Zoology, Abdul Wali Khan University Mardan, Mardan 23200, Pakistan; zahidhafsa77@gmail.com; 2 King Abdulaziz City for Science and Technology, Riyadh 12354, Saudi Arabia; asn1950r@gmail.com; 3 Department of Pharmacology and Toxicology, College of Pharmacy, King Saud University, Riyadh 11451, Saudi Arabia; mmalmutairi@ksu.edu.sa; 4 Department of Chemistry, Abdul Wali Khan University Mardan, Mardan 23200, Pakistan; m.ateeq@awkum.edu.pk; 5 Laboratory of Infectious Diseases, Joint Faculty of Veterinary Medicine, Kagoshima University, Kagoshima 890-0065, Japan; k6199431@kadai.jp; 6 Department of Emergency Medicine, Ditmanson Medical Foundation Chia-Yi Christian Hospital, Chiayi 60002, Taiwan; 7 Department of Pathology, Ditmanson Medical Foundation Chia-Yi Christian Hospital, Chiayi 60002, Taiwan; hlmarkc@gmail.com; 8 Department of Cosmetic Science, Chia Nan University of Pharmacy and Science, Tainan 717, Taiwan; 9 Ph.D. Program in Translational Medicine, Rong Hsing Research Center for Translational Medicine, National Chung Hsing University, Taichung 402, Taiwan; 10 Department of Biotechnology and Bioindustry Sciences, College of Bioscience and Biotechnology, National Cheng Kung University, Tainan 701, Taiwan

**Keywords:** *Borrelia anserina*, *Argas persicus*, domestic fowls, duck, relapsing fever, Pakistan

## Abstract

**Simple Summary:**

Soft ticks are well-known for vectoring several disease-causing pathogens that are distributed throughout the world. Surveillance of disease-causing agents associated with these ticks is important to avoid any zoonotic consequences. This study reported the epidemiology and molecular characterization of *Borrelia anserina* in *Argas persicus* collected from domestic fowls, ducks and their shelters and *Carios vespertilionis* ticks infesting bats in Khyber Pakhtunkhwa (KP), Pakistan. In the phylogenetic tree, the obtained sequences of *A. persicus* and *C. vespertilionis* clustered with the sequences from Pakistan and neighboring countries, while the Borrelial *flaB* sequence revealed its relationship with the corresponding species belonging to the relapsing fever group. Further studies are encouraged to screen soft ticks for pathogens that affect public and veterinary health.

**Abstract:**

Argasid ticks have the vectorial potential for transmitting disease-causing pathogens to avian hosts, resulting in economic losses that may not be fully estimated. *Borrelia* species are the responsible agents of borreliosis in poultry, animals and humans. Our previous studies have reported a high prevalence of *Argas persicus* infesting domestic fowls in Khyber Pakhtunkhwa (KP), Pakistan. However, molecular screening and genetic characterization of *Borrelia* spp. in *A. persicus* have been neglected in Pakistan. In this study, we focused on the molecular epidemiology and genetic characterization of *Borrelia* spp. associated with *A. persicus* ticks infesting domestic fowls and ducks, and *Carios vespertilionis* infesting bats in selected districts of KP. Overall, 1818 ticks, including females (415; 23%), males (345; 19%), nymphs (475; 26%) and larvae (583; 32%), were collected from 27 locations in nine districts (Peshawar, Mardan, Swabi, Charsadda, Chitral, Lakki Marwat, Bannu, Bajaur and Hangu) from domestic fowls, ducks and their shelters, and bats. A subset of 197 ticks was selected for DNA extraction and PCR to amplify fragments of the cytochrome c oxidase (*cox*) gene for ticks and flagellin B (*flaB*) for the detection and genetic characterization of associated *Borrelia* spp. Among these, only *Borrelia anserina* DNA was detected in 40 ticks (27.2%) of different life stages, where highest prevalence was found in female ticks (18; 45%), followed by nymphs (12; 30%), larvae (7; 17.5%) and males (3; 7.5%). Tick infestation in shelters (1081; 77%) was higher than on hosts (323; 23%). The resultant *cox* amplicons of *A. persicus* showed 100% identity with the same species reported from Pakistan, China, Iran, Kenya, Kazakhstan, Algeria and Egypt and *C. vespertilionis* show 100% identity with the species reported from Pakistan, China, Japan, Kenya, Vietnam, Spain, Netherlands, the United Kingdom and Hungry, and clustered with the aforementioned species in the phylogenetic tree. The obtained *Borrelia* sequences showed 100% identity with *B. anserina* and revealed a close resemblance to the relapsing fever group and clustered in a monophyletic clade with *B. anserina* from India, Iran and Brazil in a phylogenetic tree. These results establish the first molecular characterization of *B. anserina* in *A. persicus* infesting domestic fowls and ducks in the region, as well as their shelters. To effectively control zoonotic consequences, country-wide surveillance research should be encouraged to screen soft ticks infesting various birds for associated pathogens.

## 1. Introduction

Soft tick *Argas persicus* is the most common ectoparasite of birds that harbors disease-causing agents, including *Borrelia* spp. [1,2,3], causing diseases and un-estimated economic losses to the poultry industry. The occurrences and re-occurrences of infections triggered by bat-associated pathogens have considerably increased during the last decades and has attracted attention towards the screening of ectoparasites such as the bat tick *Carios vespertilionis* [4].

*Argas persicus* ticks infest birds such as domestic fowls (*Gallus gallus domesticus*), ducks (*Anas platyrhynchos*), turkeys (*Meleagris gallopavo*), geese (*Anser anser domesticus*), peacocks (*Pavo cristatus*), pigeons (*Columba livia*) and other wild birds [3,5,6,7]. The Gram-negative bacterium *Borrelia anserina* has been detected in *A. persicus* ticks that cause avian borreliosis in domestic fowls, doves, turkeys, geese, pheasants and canary birds in tropical and subtropical regions [3,7,8,9]. Borreliosis caused by *B. anserina* was reported for the first time in Russia in a widespread outbreak in geese [10]. After a few years, the role of *Argas* ticks as a natural vector for borreliosis was confirmed in other continents [11]. Subsequently, this pathogen attracted attention to its molecular epidemiology and the role of *Argas* ticks as vector. The role of *C. vespertilionis* as a carrier of pathogens such as viruses, bacteria and protozoans has been documented previously [12]. Among bacterial pathogens, this tick is a competent vector for *Borrelia burgdorferi* which causes Lyme disease in humans [12,13].

The members of *Borrelia* spp. complex are recognized as causative agents of numerous human and animal diseases, such as Lyme disease (LD) and relapsing fever group (RFG) [7]. The genus *Borrelia* is comprised of about ~52 species with worldwide distribution [14,15]. These species are transmitted to the host by tick species belonging to different genera, such as *Argas*, *Ornithodoros*, *Ixodes*, *Amblyomma*, *Hyalomma*, *Rhipicephalus* and *Bothriocroton*, while one species is known to be transmitted by *Pediculus humanus corporis* (human body lice) [16,17]. Borreliosis is categorized into different well-defined groups: LD, RFG borreliosis and reptile-associated group (REP) [18,19]. The accurate identification of *Borrelia* spp. at a species level is complicated by using microscopy. However, some reports differentiate the isolates of *Borrelia* spp. by using different techniques such as serological tests, immunological assays and molecular approaches [20,21,22].

Pakistan is one of the world’s largest poultry production industry, providing about 1163 million broilers. The poultry sector offers employment to more than 1.5 million people, and the investment is about Rs 750 billion to the country (Pakistan Economic Survey, Ministry of Finance, Government of Pakistan, 2022). The poultry sector faces severe issues due to vector-borne diseases, including borreliosis. *Argas persicus* ticks are reservoir hosts for disease-causing pathogens such as *Borrelia* spp. that cause borreliosis worldwide [23,24]. To reduce the economic losses of the poultry sector, regular surveillance and genetic characterization of ticks and tick-borne *Borrelia* spp. are of great importance.

To our knowledge, nine soft tick species have been reported from Pakistan. Among which five species (*Argas* sp. “*rousetti*”, *Argas persicus*, *C. vespertilionis* and *Ornithodoros* sp.) have been molecularly characterized, while four species (*Argas abdussalami*, *Argas lahorensis*, *Ornnithodoros papillipies* and *Ornithodoros tholozani)* were identified morphologically [2,25,26,27,28]. Several studies have shown a wide range of pathogens associated with *A. persicus* [3,9]. In a previous study [2], we reported the life cycle and molecular phylogeny of the fowl tick *A. persicus*. The hard ticks and associated pathogens have been investigated in Pakistan [29,30,31,32,33]. However, studies have neglected to characterize *Borrelia* spp. associated with the soft tick. This study was designed to investigate the molecular epidemiology and genetic characterization of *A. persicus* infesting domestic fowls and ducks, *C. vespertilionis* infesting bats and their associated *Borrelia* spp. in Pakistan.

## 2. Materials and Methods

### 2.1. Collection Sites

**Figure 1:**
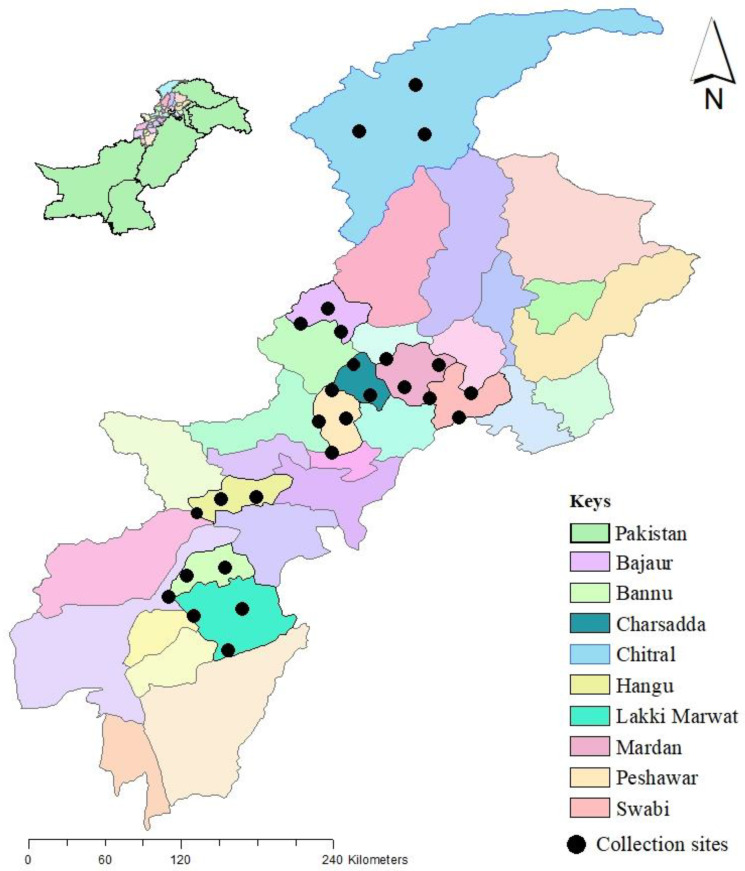
Map showing the collection sites of ticks in Khyber Pakhtunkhwa Pakistan.

The current study was carried out in nine districts of Khyber Pakhtunkhwa (KP), Pakistan, including Peshawar (34°1′33.3012″ N, 71°33′36.4860″ E), Mardan (34°12′22.0428″ N, 72°1′47.2800″ E), Swabi (34°7′12.5580″ N, 72°28′12.5544″ E), Charsadda (34°10′00.1″ N 71°45′20.0″ E), Chitral (35°45′57.9″ N 71°47′09.2″ E), Lakki Marwat (32°36′43.1″ N 70°54′09.8″ E), Bannu (32°59′27.9″ N 70°38′48.3″ E), Bajaur (34°47′30.4″ N 71°30′13.9″ E) and Hangu (33°32′06.0″ N 71°04′03.1″ E). These regions were selected based on their different climatic and geographic conditions. Three regions were selected for collection in each district. Google maps were used to collect the geographic coordinates, and the collected data were arranged in a Microsoft Excel (Microsoft Corp., Redmond, WA, USA) worksheet to construct a distribution map for the study area using ArcGIS 10.3.1 (ESRI, Redlands, CA, USA) (Figure 1).

### 2.2. Ethical Approval 

Prior approval for this research was received from the members of Advance Studies and Research Board, Abdul Wali Khan University, Mardan, under the approval no (Dir/A&R/AWKUM/2022/9395). Written/oral consent was taken from the holders of domestic fowls and ducks before tick collection from the shelters.

### 2.3. Tick Collection, Preservations and Identification

Ticks were collected from shelters of domestic fowls and ducks from different collection spots of the selected districts. Bats were captured by local farmers in their gardens using hand nets and kept in separate perforated bags. Ticks were removed carefully from the bat’s body by using fine sterile tweezers. Collected ticks were kept in sterile bottles and labeled with related information (host, collection date, coordinates, temperature and humidity). Before further analysis, ticks were washed with distilled water followed by 70% ethanol and preserved in 100% ethanol in 1.5 mL tubes. Morphological identification was carried out under the stereomicroscope (StereoBlue-euromex, Arnhem, The Netherlands) using available morphological keys [34,35].

### 2.4. Molecular Analyses 

Among the collected ticks, 147 partially fed *A. persicus* ticks (16 ticks from each district; 4 ticks from each life stage, 2 from a host and 2 from a shelter) and 3 ticks from each (F/M/N) of the ducks were randomly selected for further molecular analysis. In case of the bat collected ticks, we selected ten *C. vespertilionis* ticks from each district for the extraction of genomic DNA via standard phenol-chloroform method [36]. Sterile needles were used to make holes in ticks before DNA extraction in the 1.5 mL tube and dried in the incubator to evaporate the ethanol. The quantification of extracted DNA was performed by a NanoDrop (Nano-Q, Optizen, Daejeon, Republic of Korea). 

The extracted DNA was subjected to PCR for the molecular identification of ticks using the *cox* and 16S rRNA gene fragment. The PCR mixture (20 µL) was comprised of 1 µL each primer (10 µM), 4 µL PCR grade water, 2 µL template DNA (50–100 ng/µL) and 12 µL of Dream*Taq* PCR MasterMix (2×) (Thermo Scientific, Waltham, MA, USA). PCR for the *cox* and 16S rRNA gene was performed according to the previously described conditions [2,32]. The electrophoreses of PCR products was performed in 1.5% agarose gel stained with ethidium bromide and visualized under UV in the Gel Documentation System (UVP BioDoc-It Imaging System, Upland, CA, USA).

Each of the extracted DNA samples were screened for tick-associated *Borrelia* spp. by targeting the amplification of the *flaB* partial gene. In the initial PCR, the primer pair (Fla LL, and Fla RL) was followed by a nested PCR using 0.5 µL PCR product as the template and primer (Fla SS and Fla RS) to amplify 665 bp and 354 bp, respectively (Table 1). The PCR conditions for both reactions were set according to Stromdahl et al. [37]. In the PCR reactions, a *Borrelia* spp. of *Amblyomma gervaisi*, *Rickettsia massiliae* of *Rhipicephalus microplus*, *Ehrlichia* spp. and distilled water were taken as a positive and negative control, respectively.

### 2.5. Sequence and Phylogenetic Analysis

The amplified PCR products showing the expected size were purified using a commercial NucleoSpin Gel and PCR Clean-up Kit (Macherey-Nagel, Duren, Germany) following the manufacturer’s directions. The purified PCR amplicons were sent to a commercial company for bidirectional sequencing (Macrogen, Inc., Seoul, Republic of Korea). The obtained sequences were subjected to trimming and assembled to remove the primer’s contamination and poor reading regions in SeqMan v. 5.0 (DNASTAR, Inc., Madison, WI, USA). Trimmed sequences were subjected to BLAST (Basic Local Alignment Search Tool) at NCBI (National Center for Biotechnology Information) [41] to download identical sequences. Identical sequences were downloaded and aligned by ClustalW Multiple alignments [42] in BioEdit alignment editor v. 7.0.5 (Raleigh, NC, USA) [43]. The phylogenetic tree, based on *cox* for ticks and *flaB* sequence for *Borrelia* spp., was constructed using the Maximum Likelihood method based on the Kimura 2-parameter in MEGA-X (Molecular evolutionary genetics analysis), aligned by MUSCLE [44] using 1000 bootstrap replicons [45].

### 2.6. Statistical Analyses 

The data such as tick infestation on hosts and shelters in the different regions were assembled and arranged in spreadsheets on Microsoft Excel v. 2016. The tick infestation of each life stage on host and shelters, and prevalence of *B. anserina* between different regions, was analyzed in GraphPad Prism v. 5.00 (GraphPad Software Inc., San Diego, CA, USA).

## 3. Results

### 3.1. Tick Infestation 

All the collected ticks were morphologically identified as *A. persicus* based on the presence of a lateral line and possessed less than 100 integumental cells around the body margin [30]. Among 1818 collected ticks, 415 (23.00%) were females, 345 (19.00%) were males, 475 (26.00%) were nymphs and 583 (32.00%) were larvae from nine districts (Peshawar, Mardan, Swabi, Charsadda, Chitral, Lakki Marwat, Bannu, Bajaur and Hangu) (Table 2). The larval stage were found the highest most, while the male ticks were found the least. Overall, the highest prevalence of ticks was found in the Mardan district (297; 16%) followed by Peshawar (292; 16.3%), Charsadda (283; 15.6%), Swabi (234; 13%), Hangu (173; 9.5%) Lakki Marwat (169; 9.3%), Bannu (159; 8.7%), while the lowest prevalence was found in the districts Bajaur (112; 6.2%) and Chitral (99; 5.4%).

Among the different districts, the highest tick prevalence of *A. persicus* was found in Peshawar (201; 14.31%), followed by Mardan (199; 14.17%), Charsadda (194; 13.81%), Swabi (173; 12.32%), Lakki Marwat (169; 12.03%), Bannu (159; 11.32%) and Bajaur (112; 7.97%), while the lowest infestation was found in the district Hangu (98; 6.98%) and Chitral (58; 4.13%), while tick prevalence on ducks was (41; 2.2%). Tick-infested ducks were only found in the Chitral district. The *C. vespertilionis* were collected from five districts, in which the highest prevalence was found in the Mardan district (98; 23.6%) followed by Peshawar (91; 22), Charsadda (89; 21.4%), Hangu (75; 18%) and Swabi (61; 15%). Tick infestation of each life stage between hosts and shelters is shown in Table 2.

In a total of 189 examined shelters, tick infestation was found in 79 (42%) shelters in different selected districts. Among the selected districts, the highest prevalence of infested shelters was found in the Charsadda district (20/12; 55%), followed by Peshawar (25/12; 48%), Mardan (23/11; 47.82%), Swabi (21/10; 47.61%), Bannu (20/9; 45%) and Hangu (12/5; 41.66%). While the lowest infestation was found in the Chitral district (21/5; 23.80%), Lakki Marwat (21/7; 33.33%) and Bajaur (18/7; 38.88%). Among the eight visited shelters of ducks in Chitral, only two (25%) were tick-infested.

### 3.2. Detection of Borrelia anserina 

Among a subset of 197 screened ticks for *Borrelia* spp., 40 (27.2%) were found positive for *B. anserina* based on *flaB* fragment (long and short sequences). Among the 40 positive samples, only 5 showed faint bands in the initial PCR (long fragments), while 35 were positive in nested PCR (short fragments) and sequencing. In 40 (27.2%) *Borrelia* positive ticks, the highest prevalence was found in female ticks (18/40; 45%), followed by nymphs (12/40; 30%), larvae (7/40; 17.5%) and males (3/40; 7.5%). The prevalence of ticks, infested shelters, life stages and details of *B. anserina* detection on host or shelter-collected ticks from selected districts are shown in Table 2. In the case of *C. vespertilionis*, all the screened ticks were found to be negative for *B. anserina.* Both *A. persicus* and *C. vespertilionis* were found to be negative for the presence of *Rickettsia* and *Ehrlichia* DNA. 

### 3.3. Molecular and Phylogenetic Analysis

**Figure 2:**
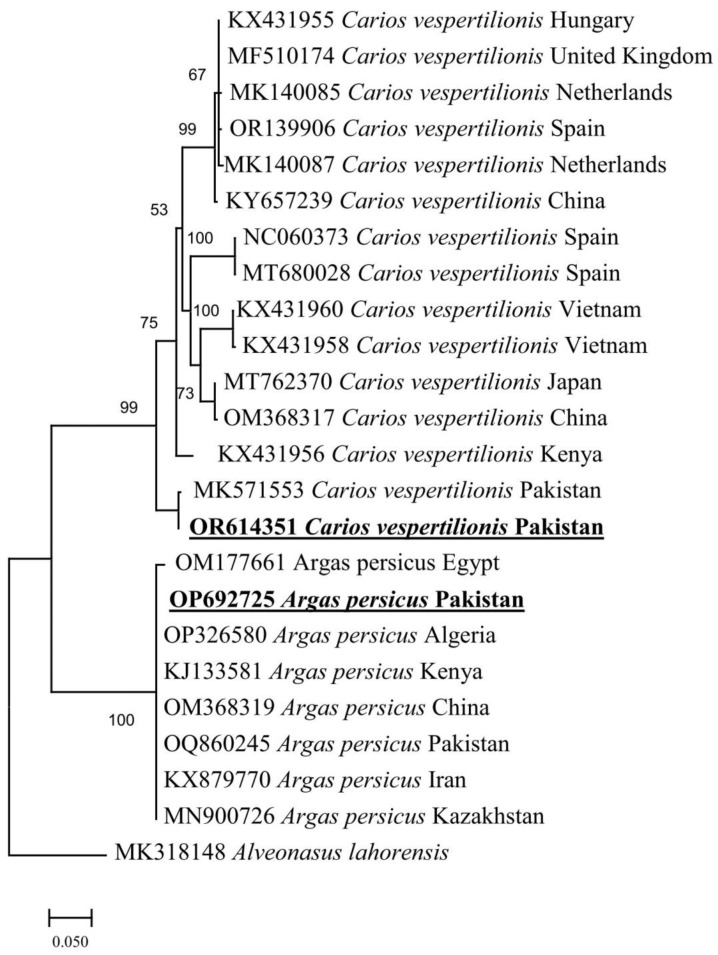
Maximum likelihood phylogenetic tree based on *cox* sequences of *Argas persicus* and *Carios vespertilionis*. *Alveonasus lahorensis* was taken as an outgroup. GenBank accession numbers are followed by species (italic) and country names. The obtained *A. persicus* and *C. vespertilionis* sequences are represented in bold and underlined.

The BLAST results of the obtained cox sequences of ticks showed 100% identity with *A. persicus* from Pakistan-OQ860245, China-OM368319, Kenya-KJ133581, Algeria-OP326580 Kazakhstan-MN900726, Iran-KX879770 and Egypt-OM177661, and C. vespertilionis showed 100% identity with same species from Pakistan-MK571553, China-OM368317, Japan-MT762370, Kenya-KX431956, Vietnam-KX431960, KX431958, Netherlands-MK140085, MK140087, China-KY657239, Spain-NC060373, MT680028, OR139906, the United Kingdom-MF510174 and Hungary-KX431955. In the phylogenatic tree, the obtained sequences were clustered with mentioned sequences (Figure 2). The consensus sequences of *A. persicus* and *C. vespertilionis* were uploaded to GenBank (OP692725, OR614351).

**Figure 3:**
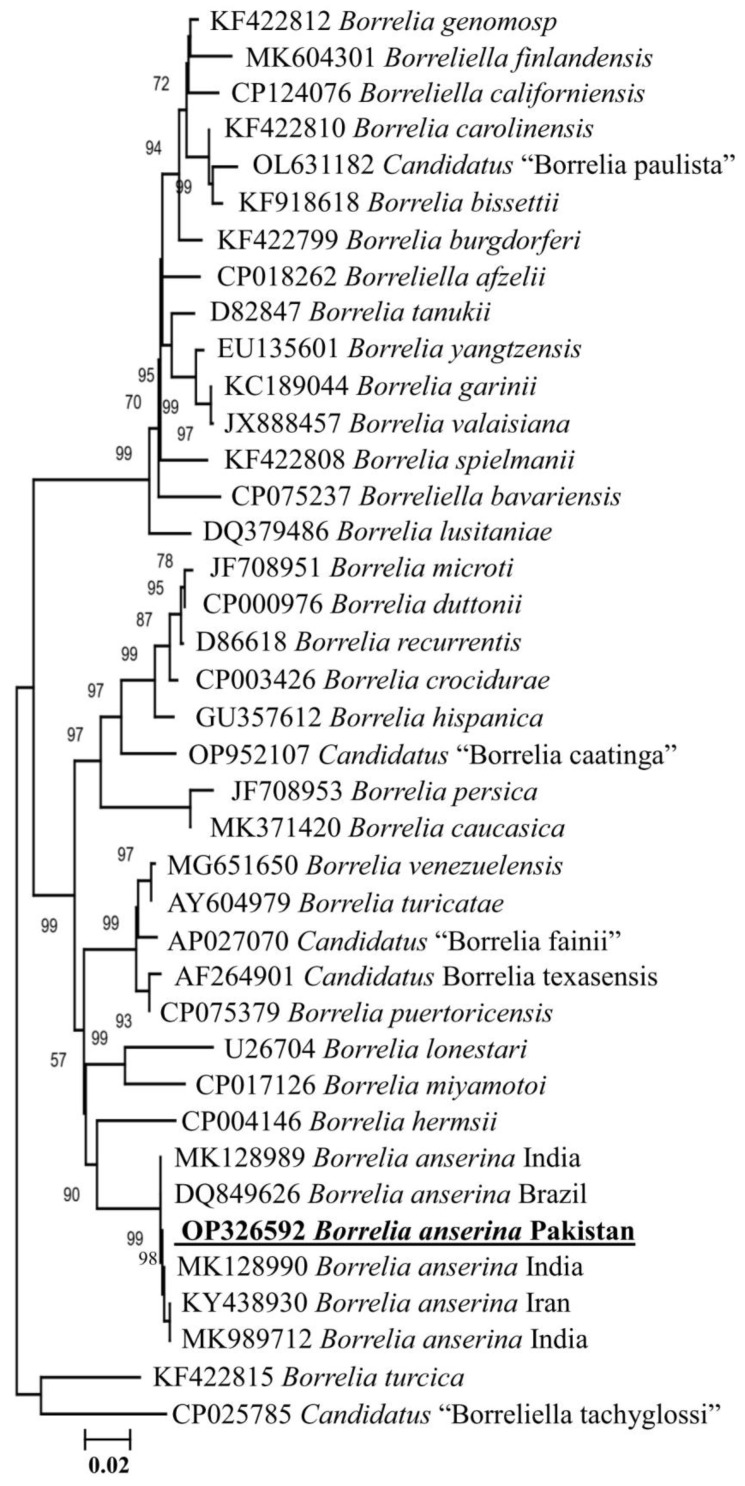
Maximum likelihood phylogenetic tree based on *flaB* sequence of *Borrelia anserina*. *Borrelia turcica* and “*Candidatus* Borrelia tachyglossi” were taken as outgroups. GenBank accession numbers are followed by species (italic) and country names (in the case of *B. anserina*). The obtained *B. anserina* sequence are represented in bold and underlined.

The amplified *flaB* fragments showed 100% identity to the sequences of *B. anserina* from India-MK989712, MK128989, MK128990, Iran-KY438930 and Brazil-DQ849626. Among the 40 *B. anserina* sequences (5 long and 35 short), both long and short sequences showed identity to each other. Thus, one consensus sequence from each long and short sequence of *B. anserina* was uploaded to GenBank (OP326592, ON148464). The obtained sequence of *B. anserina* was clustered with the RFG species in the phylogenetic tree (Figure 3).

## 4. Discussion

Soft ticks *A. persicus* and *C. vespertilionis* are the common ectoparasites of several birds and bats, respectively, that have been morphologically and genetically characterized in several countries, including Pakistan [2,25,28,46,47,48]. However, knowledge of the associated *Borrelia* spp. has not been molecularly explored in the region. To fill this knowledge gap, the collected ticks were morphologically and molecularly examined and confirmed as *A. persicus* and *C. vespertilionis* infesting domestic fowls, ducks and bats in nine districts of KP, Pakistan. To our knowledge, this is the first report on the molecular epidemiology, genetic characterization, and comparison of *Borrelia* spp. on host- (domestic fowls and ducks) and shelter-collected *A. persicus* ticks in Pakistan. Ticks were screened for spirochetes, and *B. anserina* DNA was detected in *A. persicus* ticks while *C. vespertilionis* ticks were found negative for *Borrelia* DNA. The phylogenetic analysis of tick *cox* sequences revealed their closest relationship with the same species reported from Pakistan [2,28]. The phylogenetic tree of *Borrelia flaB* revealed that the aforementioned species belongs to the RFG of *Borrelia* species. The collected ticks were also screened for the presence of *Rickettsia* and *Ehrlichia;* however, DNA of none of these agents was detected.

The climatic conditions are mostly intricate with the prevalence and diversity of ticks in a region [49,50]. The highest prevalence of *A. persicus* was reported in hot atmospheric and humid regions such as Peshawar and Mardan, while the lowest prevalence was in low temperature, high altitude and less humid regions like Bajaur and Chitral, as previously described [2], while *C. vespertilionis* was most prevalent in the Mardan district and least prevalent in the Swabi district. The variation in prevalence with environmental temperature, high altitude, and rainfall indicates that the ticks prefer high temperature and rich humid regions where they can survive and reproduce favorably [3,32,50]. The annual increase in the climatic temperature facilitates the tick’s survival in the least prevalent and tick-free regions [51].

Complexities in the morphological identification of Argasid ticks still exist for taxonomists [52]. Thus, molecular approaches are used for accurate identification and genetic characterization of different species, especially Argasid ticks [23,53,54,55,56]. The host- (domestic fowls) and shelter-collected ticks were molecularly confirmed as a species, *A. persicus*, with the bat-collected ticks as *C. vespertilionis*. The results revealed that the ticks inhabiting shelters may pose severe health threats to animals and humans due to accidental infestation and the capability of transmitting pathogens [57,58,59]. The molecular data showed a close identity of the collected ticks with the previously reported *A. persicus* and *C. vespertilionis* from the same region. In the phylogenetic tree, the obtained *cox* sequences of ticks clustered with the same species reported from Pakistan and neighboring countries which is in agreement with the previous studies [2,28,54]. 

*Argas persicus* ticks are commonly involved in the transmission of *Borrelia* spp. in various domestic and wild birds [3]. The DNA of *flaB* for *B. anserina* was detected in all life stages, like larvae, nymphs, males and females of *A. persicus*, indicating their transovarial transmission [17]. The host- and shelter-collected ticks were comparatively screened and found positive for *B. anserina*, while in a similar screening, *C. vespertilionis* was found negative for any *Borrelia* spp. In the phylogenetic tree, *B. anserina* based on *flaB* sequences grouped with *Borrelia* species belongs to RFG. The outcomes of our study are consistent with the findings of previous reports that demonstrated *B. anserina* falls within a monophyletic clade with the species of the RFG [16,55,56,60,61,62,63,64,65]. The presence of *Borrelia* spp. has also been reported in several birds such as domestic fowls, ducks, geese, king penguins, blackbirds and nightingales in different regions of the world [52,53]. Birds should be investigated for *Borrelia* spp. to restrict any *Borrelia* epidemics and zoonosis consequences. Since this study represents a limited number of analyzed specimens from selected regions, detailed large-scale molecular surveillance of soft tick-associated pathogens is regularly needed to avoid zoonotic threats to birds and humans.

## 5. Conclusions

This study for the first time reported the genetic characterization of *B. anserina* in *A. persicus* infesting domestic fowls and ducks in KP, Pakistan. Additionally, the molecular data of *C. vespertilionis* revealed its closed resemblance with a previously reported tick from Pakistan. *B. anserina* was detected and genetically characterized for the first time in both the host- and shelter-collected *A. persicus* ticks. These results suggest that the freely moving infected *A. persicus* ticks in the shelters may pose health threats to healthy birds and humans. In the phylogenetic tree, the *flaB* sequences of *B. anserina* clustered with the corresponding species belonging to the RFG. Further studies are highly encouraged to screen soft ticks for *Borrelia* and other pathogens that affect public and veterinary health. 

## Figures and Tables

**Table 1:** List of primers used for the amplification of ticks and their associated pathogens.

S#	Organism	Gene	Primers Sequence (5′-3′)	Amplicons Size	Reference
1	Tick	*cox*	HC02198: TAAACTTCAGGGTGACCAAAAAATCA LCO1490: GGTCAACAAATCATAAAGATATTG G	710 bp	[38]
2	Tick	16S	TTTGGGACAAGAAGACCCTATGAATTT ACATCGAGGTCGCAATCAATTTTATC	250 bp	[2]
3	*Borrelia*	Fla LL Fla RL Fla SS Fla RS	ACATATTCAGATGCAGACAGAGGT GCAATCATAGCCATTGCAGATTGT AACAGCTGAAGAGCTTGGAATG CTTTGATCACTTATCATTCTAATAGC	665 bp 354 bp	[37]
4	*Rickettsia*	*gltA*	CS-78: GCAAGTATCGGTGAGGATGTAAT CS-323: GCTTCCTTAAAATTCAATAAATCAGAT	401 bp	[39]
5	*Ehrlichia*	16S	EHR16SD: GGTACCYACAGAAGAAGTCC EHR16SR: TGCACTCATCGTTTACAG	344 bp	[40]

**Table 2:** *Borrelia anserina* detected in different life stages of the *Argas persicus* collected in nine districts, KP, Pakistan.

Districts	Tick Species	Host	Observed/Infested (%)	No. of Collected Ticks F, M, N, L (n; %)	Detection of *Borrelia anserina* F, M, N, L	Total (%)
Peshawar	*Argas persicus*	Shelters	25/12 (48)	45, 40, 29, 17 (131; 7.2)	2, 1, 1, 0	4 (10)
		Domestic fowls	167/99 (59)	9, 7, 23, 31 (70; 3.9)	1, 0, 0, 1	2 (5)
	*Carios vespertilionis*	Bats	5/1 (40)	1, 2, 8, 80 (91; 5.0)	0	0
Mardan	*A. persicus*	Shelters	23/11 (47.82)	47, 44, 41, 15 (147; 8)	1, 0, 1, 0	2 (5)
		Domestic fowls	145/89 (61)	7, 6, 17, 22 (52; 3)	1, 0, 1, 1	3 (7.5)
	*C. vespertilionis*	Bats	6/1 (50)	2, 3, 6, 87 (98; 5.4)	0	0
Swabi	*A. persicus*	Shelters	21/10 (47.61)	40, 33, 39, 12 (124; 6.8)	1, 0, 1, 0	2 (5)
		Domestic fowls	134/99 (74)	6, 5, 5, 33 (49; 2.7)	1, 0, 0, 0	1 (2.5)
	*C. vespertilionis*	Bats	5/1 (40)	0, 0, 3, 58 (61; 3.3)	0	0
Charsadda	*A. persicus*	Shelters	20/12 (55)	51, 45, 53, 13 (162; 9)	1, 0, 1, 0	2 (5)
		Domestic fowls	116/91 (78)	4, 3, 14, 11 (32; 1.8)	0, 0, 0, 1	1 (2.5)
	*C. vespertilionis*	Bats	7/2 (43)	4, 3, 12, 70 (89; 5)	0	0
Chitral	*A. persicus*	Shelters	21/5 (23.80)	11, 9, 21, 3 (44; 2.4)	1, 0, 0, 1	2 (5)
		Domestic fowls	142/45 (32)	2, 3, 3, 6 (14; 1)	1, 1, 1, 0	3 (7.5)
	*C. vespertilionis*	Bats	0	0	0	0
	*A. persicus*	Ducks	54/19 (35)	12, 16, 13, 0 (41; 2.2)	1, 0, 0, 0	1 (2.5)
Lakki Marwat	*A. persicus*	Shelters	21/7 (33.33)	45, 33, 53, 9 (140; 7.7)	1, 0, 0, 0	2 (5)
		Domestic fowls	112/69 (61)	6, 3, 6, 14 (29; 1.6)	1, 0, 1, 1	2 (5)
	*C. vespertilionis*	Bats	0	0	0	0
Bannu	*A. persicus*	Shelters	20/9 (45)	42, 32, 45, 11 (130; 7)	0, 0, 1, 1	2 (5)
		Domestic fowls	121/66 (55)	5, 4, 5, 15 (29; 1.6)	1, 0, 1, 0	2 (5)
	*C. vespertilionis*	Bats	0	0	0	0
Bajaur	*A. persicus*	Shelters	18/7 (38.88)	35, 24, 31, 3 (93; 5)	1, 1, 0, 0	2 (5)
		Domestic fowls	103/59 (57)	5, 2, 5, 7 (19; 1)	1, 0, 1, 1	3 (7.5)
	*C. vespertilionis*	Bats	0	0	0	0
Hangu	*A. persicus*	Shelters	12/5 (41.66)	30, 23, 27, 0 (80; 4.4)	1, 0, 0, 1	2 (5)
		Domestic fowls	111/46 (41)	4, 3, 7, 4 (18; 1)	1, 0, 1, 0	2 (5)
	*C. vespertilionis*	Bats	4/2 (5)	2, 2, 9, 62 (75; 4)	0	0
Total	*A. persicus*	Shelters	189/79 (42)	358, 293, 347, 83 (1081; 59.4)	10, 2, 5, 3	20 (13.6)
		Domestic fowls	1205/682 (57)	48, 42, 90, 143 (323; 17.8)	8, 1, 7, 4	20 (13.6)
	*C. vespertilionis*	Bats	27/7 (26)	9, 10, 38, 357 (414; 22.8)	0	0
Total				415, 345, 475, 583 (1818)	18, 3, 12, 7	40

F: female, M: male, N: nymph, L: larvae.

## Data Availability

All data generated or analyzed during this study were included in this article. Further inquiries can be directed to the corresponding author.
